# Artificial Intelligence for Contrast-Free MRI: Scar Assessment in Myocardial Infarction Using Deep Learning–Based Virtual Native Enhancement

**DOI:** 10.1161/CIRCULATIONAHA.122.060137

**Published:** 2022-09-20

**Authors:** Qiang Zhang, Matthew K. Burrage, Mayooran Shanmuganathan, Ricardo A. Gonzales, Elena Lukaschuk, Katharine E. Thomas, Rebecca Mills, Joana Leal Pelado, Chrysovalantou Nikolaidou, Iulia A. Popescu, Yung P. Lee, Xinheng Zhang, Rohan Dharmakumar, Saul G. Myerson, Oliver Rider, Keith M. Channon, Stefan Neubauer, Stefan K. Piechnik, Vanessa M. Ferreira

**Affiliations:** Oxford Centre for Clinical Magnetic Resonance Research (Q.Z., M.K.B., M.S., R.A.G., E.L., K.E.T., R.M., J.L.P., C.N., I.A.P., Y.P.L., S.G.M., O.R., S.N., S.K.P., V.M.F.), Radcliffe Department of Medicine, University of Oxford, United Kingdom.; Division of Cardiovascular Medicine (Q.Z., M.K.B., M.S., R.A.G., E.L., K.E.T., R.M., J.L.P., C.N., I.A.P., Y.P.L., S.G.M., O.R., K.M.C., S.N., S.K.P., V.M.F.), Radcliffe Department of Medicine, University of Oxford, United Kingdom.; Krannert Cardiovascular Research Center, Indiana School of Medicine/IU Health Cardiovascular Institute, Indianapolis (X.Z., R.D.).; Department of Bioengineering, University of California in Los Angeles (X.Z.).; Faculty of Medicine, University of Queensland, Brisbane, Australia (M.K.B.).

**Keywords:** artificial intelligence, cicatrix, magnetic resonance imaging, myocardial infarction

## Abstract

Myocardial scars are assessed noninvasively using cardiovascular magnetic resonance late gadolinium enhancement (LGE) as an imaging gold standard. A contrast-free approach would provide many advantages, including a faster and cheaper scan without contrast-associated problems.

**Methods:**

Virtual native enhancement (VNE) is a novel technology that can produce virtual LGE-like images without the need for contrast. VNE combines cine imaging and native T1 maps to produce LGE-like images using artificial intelligence. VNE was developed for patients with previous myocardial infarction from 4271 data sets (912 patients); each data set comprises slice position-matched cine, T1 maps, and LGE images. After quality control, 3002 data sets (775 patients) were used for development and 291 data sets (68 patients) for testing. The VNE generator was trained using generative adversarial networks, using 2 adversarial discriminators to improve the image quality. The left ventricle was contoured semiautomatically. Myocardial scar volume was quantified using the full width at half maximum method. Scar transmurality was measured using the centerline chord method and visualized on bull’s-eye plots. Lesion quantification by VNE and LGE was compared using linear regression, Pearson correlation (*R*), and intraclass correlation coefficients. Proof-of-principle histopathologic comparison of VNE in a porcine model of myocardial infarction also was performed.

**Results:**

VNE provided significantly better image quality than LGE on blinded analysis by 5 independent operators on 291 data sets (all *P*<0.001). VNE correlated strongly with LGE in quantifying scar size (*R*, 0.89; intraclass correlation coefficient, 0.94) and transmurality (*R*, 0.84; intraclass correlation coefficient, 0.90) in 66 patients (277 test data sets). Two cardiovascular magnetic resonance experts reviewed all test image slices and reported an overall accuracy of 84% for VNE in detecting scars when compared with LGE, with specificity of 100% and sensitivity of 77%. VNE also showed excellent visuospatial agreement with histopathology in 2 cases of a porcine model of myocardial infarction.

**Conclusions:**

VNE demonstrated high agreement with LGE cardiovascular magnetic resonance for myocardial scar assessment in patients with previous myocardial infarction in visuospatial distribution and lesion quantification with superior image quality. VNE is a potentially transformative artificial intelligence–based technology with promise in reducing scan times and costs, increasing clinical throughput, and improving the accessibility of cardiovascular magnetic resonance in the near future.

**Clinical Perspective**

**What Is New?**

- Artificial intelligence–based virtual native enhancement (VNE) in cardiac magnetic resonance imaging can reliably detect and visualize myocardial scar in patients with a history of previous myocardial infarction.
- Developed on 4271 data sets from 912 patients, VNE demonstrated superior image quality compared with late gadolinium enhancement and correlated strongly with late gadolinium enhancement in quantifying scar size and transmurality.
- VNE showed excellent visuospatial agreement with histopathology in the first proof-of-principle comparison in 2 cases of a porcine model of myocardial infarction.

**What Are the Clinical Implications?**

- VNE provides a contrast-free alternative to late gadolinium enhancement for tissue characterization in patients with previous myocardial infarction, holding promise to eliminate the need for contrast in a large proportion of cardiovascular magnetic resonance scans.
- VNE has great potential for further translation across a wide range of myocardial pathologies.
- VNE is an emerging artificial intelligence technology that can reduce scan times and costs, increase clinical throughput, and improve the accessibility of cardiovascular magnetic resonance imaging in the near future.

**Editorial, see p 1504**

Ischemic heart disease (IHD) is increasingly prevalent and a leading cause of morbidity and mortality worldwide.^1^ Myocardial scars and viability are assessed on cardiovascular magnetic resonance (CMR) imaging using late gadolinium enhancement (LGE) as the imaging gold standard and is useful to inform clinical decision-making regarding revascularization, risk stratification, and long-term prognosis in the management of patients with IHD.^2,3^ LGE-CMR is used routinely in contemporary clinical practice, offers high spatial resolution, detects small and subendocardial infarctions that may be missed by other imaging modalities,^4^ and is highly reproducible for assessment of infarct size.^5^ Landmark studies have shown that the extent of infarct size and transmurality on LGE-CMR predicts functional improvement after revascularization.^6–10^ LGE requires intravenous gadolinium-based contrast agents, which increase scan time and are contraindicated in certain populations. There is increasing interest in transitioning toward less invasive, contrast-free techniques, which can shorten scan and preparation times, reduce costs, and improve the availability of CMR.

Native (precontrast) CMR methods, such as steady-state free precession cine imaging and T1 mapping, are gadolinium-free approaches for cardiac phenotyping that can detect abnormalities associated with LGE signals.^11^ T1 mapping has shown promise in the contrast-free assessment of previous myocardial infarction (MI)^12^ with good correlation with histopathology when performing detailed quantitative image analysis.^13,14^ However, the diagnostic performance of T1 mapping using visual analysis alone is suboptimal because of a lack of standardized display of the T1 maps and other confounders.^15–17^ It would be highly advantageous to develop a means to visualize T1 maps easily in routine clinical practice.

Virtual native enhancement (VNE), a novel artificial intelligence (AI) approach, has shown promise in this regard. VNE uses deep learning to generate LGE-like images by enhancing the imaging signals in native T1 maps and cine magnetic resonance images. VNE was first validated in patients with hypertrophic cardiomyopathy, achieving excellent visuospatial agreement with standard LGE with superior image quality.^18^ VNE presents a potential solution to achieve rapid magnetic resonance imaging without the need for contrast agent administration. VNE holds promise to extend to other major pathologies encountered in clinical practice, such as assessment of myocardial scars and viability in patients with previous MI, which comprises a large proportion of routine CMR referrals.^19^

We hypothesized that further deep learning development of VNE can enable gadolinium-free visualization of myocardial scars in the assessment of MI. The VNE deep learning model was developed and tested on large-scale data sets of patients with previous MI to enable LGE-like visualization and quantification of myocardial scar burden, compared with conventional LGE, in a ready-to-use clinical format. We provide the first proof-of-principle histopathologic comparison of VNE with a porcine model of MI.

## Methods

### Materials

The anonymized test data and materials that support the findings of this study are available from the corresponding author upon reasonable request and subject to study committees’ approval.

We performed this single-center study at the University of Oxford John Radcliffe Hospital. Human CMR data sets were collected from the University of Oxford Centre for Clinical Magnetic Resonance Research clinical service (using a 1.5T Avanto or Avanto Fit scanner; Siemens Healthcare) and the OxAMI study (Oxford Acute Myocardial Infarction)^20^ (using a 3T Verio or Tim Trio scanner; Siemens Healthcare). For the clinical service data set, consecutive patient CMR clinical reports from 2010 and 2021 were screened for presence of MI on LGE by 2 experienced clinicians (M.K.B., V.M.F.). Patients were included if there was evidence of previous MI (ie, an ischemic pattern of subendocardial or transmural LGE scars), no clinical history or CMR features of acute MI (eg, substantial myocardial edema, presence of microvascular obstruction) given the effect that acute myocardial edema may have on native T1 values, no substantial concomitant myocardial pathology (ie, no underlying cardiomyopathy or infiltrative disease), and signed written consent for research. OxAMI is a single-center prospective study of patients presenting with acute MI and in follow-up during their recovery. CMR data from the follow-up time point (typically 6 months after MI) were used for this development.

All materials in this study included written informed consent from participants for research and the necessary approvals from research ethics and study committees. All participants from the clinical service included in this study had signed written consent for their anonymized data to be used for clinical research as per local unit protocols. Further ethics approval for the retrospective use of anonymized data was granted by the National Health Service Health Research Authority (Integrated Research Application System reference number 210155). Approval for the OxAMI study was provided by the Health Research Authority’s National Research Ethics Service Committee–South Central Oxford A (reference number 11/SC0397).

Once CMR data were acquired, further screening was performed to ensure that only data sets with slice position–matched native T1 map, precontrast or postcontrast cine, and LGE images were included. Quality assurance of T1 mapping sequences was performed using a standardized phantom as described previously.^21^

As per our routine clinical protocol, short-axis cines were typically acquired postcontrast. These scans were used for training the VNE generator, with the cine images transferred to synthetic precontrast images using a dedicated neural network approach (detailed in Supplemental Material 3). All patient scans with cine images acquired before gadolinium injection were identified and reserved as an independent test data set to generate VNE images that were completely free of contrast agent.

In vivo CMR scans (including cine, T1 maps using the ShMOLLI method [shortened modified look-locker inversion recovery],^22^ and phase-sensitive inversion-recovery LGE) of a porcine model of MI were performed 8 to 9 weeks after inducing an MI with ligation of the left anterior descending artery for 90 minutes followed by reperfusion at Cedars-Sinai Medical Center. The study received institutional ethics approval. Two pigs were killed 48 hours after the 8- to 9-week CMR scan to obtain histologic data of the left ventricle including ex vivo slices (5 μm thick) and magnified images taken with light microscopy after applying hematoxylin & eosin stain and Masson trichrome stain, respectively. All histopathologic slices were matched as closely as possible to the corresponding CMR images. VNE was produced offline at the Oxford center and validated against the histopathology data.

### Deep Learning Method

**Figure 1.**
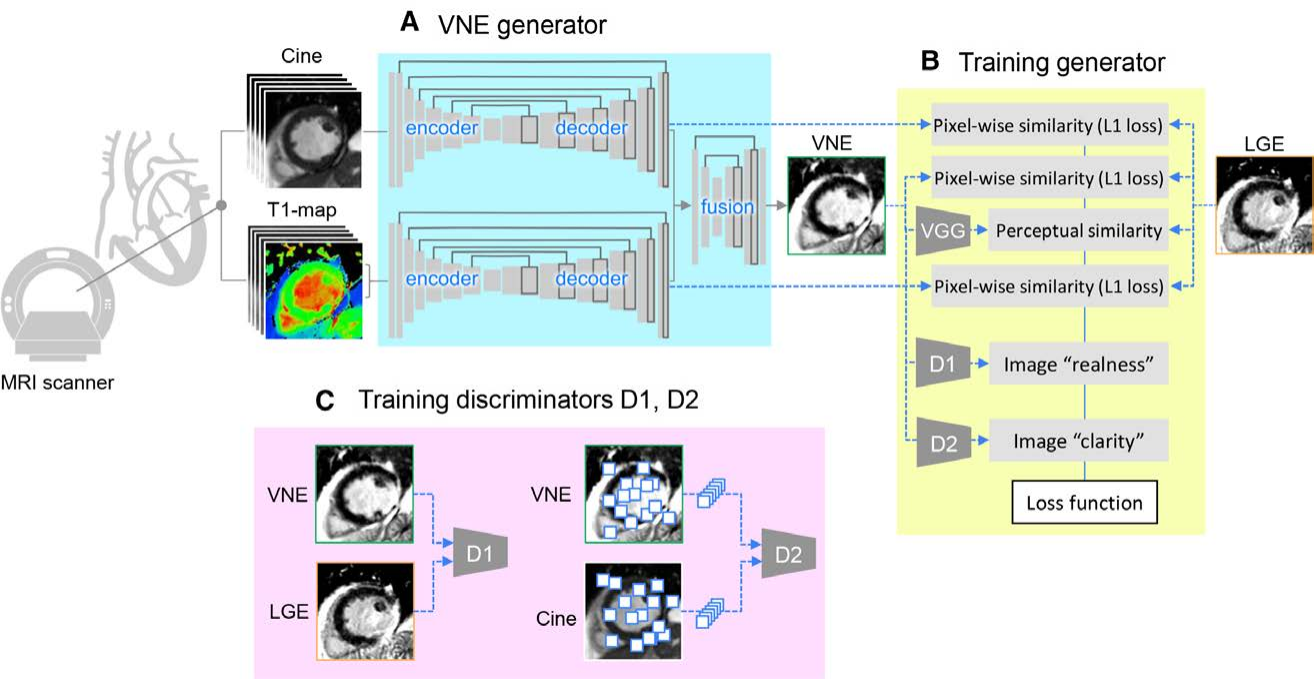
**Deep learning approach of VNE for myocardial infarction. A**, The neural network that combines cine frames and T1 maps (including inversion recovery–weighted images) and produces virtual native enhancement (VNE) images. **B**, Training VNE generator with a modified conditional generative adversarial network (cGAN) approach. **C**, Simultaneous training of 2 discriminators D1 and D2. LGE indicates late gadolinium enhancement; and MRI, magnetic resonance imaging.

The VNE generator has multiple U-Net^23^ streams to process cine frames and T1 maps (including inversion-recovery weighted images) and to produce feature maps, similar to previously described methods.^18^ The feature maps by U-Nets are concatenated and input into a further neural network block to fuse the information from multimodalities and produce a final VNE image (Figure 1A).

The neural networks were trained using a conditional generative adversarial network approach^24^ tailored for this VNE application. The generative adversarial network optimizes the generator G together with 2 adversarial discriminators D1 and D2. G was trained to produce VNE images that match LGE images in perceptual similarity^25^ (using a pretrained VGGNet^26^) and pixelwise similarity (as measured by L1 loss), resemble LGE in image appearance (supervised by D1), and have improved image clarity learned from cine images (supervised by D2; Figure 1B). D1 was trained to distinguish VNE and LGE images and D2 was trained to distinguish the clarity of low-level image features randomly sampled from VNE and cine images (Figure 1C). To improve the transparency of the deep learning decision making, an additional convolutional layer was attached at the end of the cine and T1 map streams, producing intermediate VNE signals that match LGE in pixelwise similarity. Full deep learning details are provided as Expanded Methods in Supplemental Material 2 for reproducibility.

### Image Quality Assessment and Data Analysis

Three experienced clinical operators (M.K.B., M.S., C.N.) and 2 senior CMR radiographers (R.M., J.L.P.) scored the image quality of VNE and LGE blindly, as described previously.^18^ A scale from 0 to 100 was used and guided by 5 categories: uninterpretable (score 0 to 20), poor quality (score 21 to 40), acceptable (score 41 to 60), good quality (score 61 to 80), or excellent quality (score 81 to 100). Semiautomated myocardial lesion quantification by VNE and LGE was performed as follows: epicardial and endocardial left ventricular (LV) contours were initialized automatically^27^ and corrected manually on all images by an experienced blinded operator. A remote reference region of interest without LGE and an LV blood pool region of interest were added and scar region of interest was calculated using the full width at half maximum method, taking average signal intensities of the remote myocardial and LV blood pool regions of interest as the minimum and maximum values. Myocardial scar volume fraction was quantified for each patient as the sum of the lesion area divided by the total LV myocardial area in all available short-axis slices. Two CMR experts (M.K.B., V.M.F.) independently scored the test VNE and LGE images on visuospatial agreement and myocardial scar extent, with any differences in assessment achieved by consensus.

Transmurality was measured using the centerline chord method^17^ by calculating the extent of the scar along 100 equally placed chords drawn on the LV myocardium on each slice. Distribution of the transmurality was visualized using the American Heart Association 16-segment model,^28^ with a colormap to indicate the likelihood of myocardial viability.^6^ This viability-likelihood colormap was inspired by the landmark study by Kim et al.,^6^ which showed that up to 80% of myocardial segments with <25% transmural LGE extent may gain functional recovery after revascularization, compared with <10% of segments in which infarction is >50% wall thickness. Mean scar transmurality for each patient was calculated by averaging the transmurality across all the chords on all available slices that had at least 1% of scar extent.^17^

### Statistical Analysis

Statistical data analysis was performed in Python (version 3.9.6) using SciPy (version 1.6.2) and Pingouin (version 0.4.0) packages. For image quality assessment and control, interobserver variability was calculated as standard deviation and intraclass correlation coefficient (ICC). The statistical significance of differences in VNE and LGE quality scores was analyzed using nonparametric Wilcoxon tests. Correlation between scar size and transmurality by VNE and LGE was assessed using linear least-squares regression (taking VNE as exposure variables, LGE as outcome variables), Pearson correlation coefficients, and ICC. The model ICC(3,k) was used to evaluate both the strength of correlation and the concordance.^29^ Bland-Altman analysis was performed to analyze any systematic differences between quantification by VNE and LGE. Statistical significance was defined as *P*<0.05.

## Results

### Study Population

**Figure 2.**
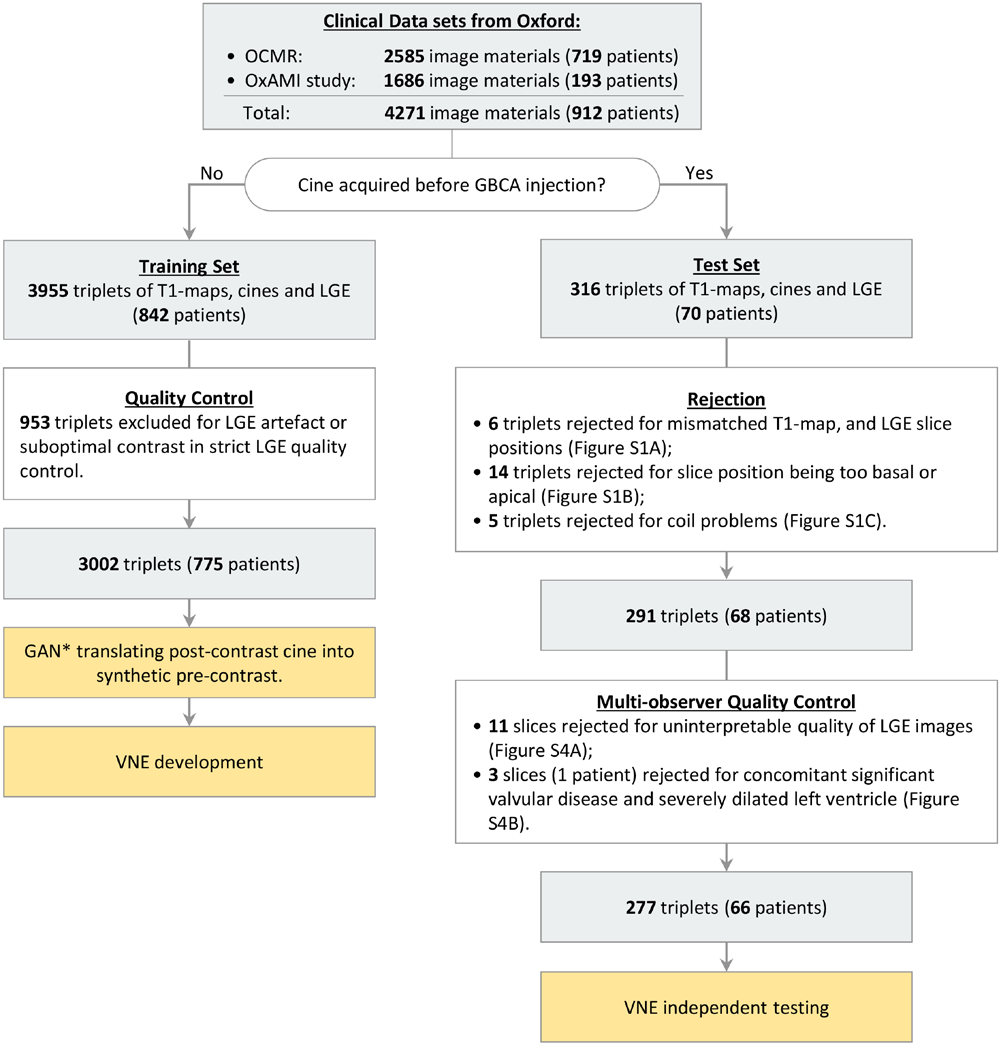
**Flow of patient selection for VNE development and testing using clinical data sets.** Clinical data sets used were from the University of Oxford Centre for Clinical Magnetic Resonance Research (OCMR) and the OxAMI study (Oxford Acute Myocardial Infarction). The training data set underwent strict late gadolinium enhancement (LGE) quality control to train the neural network to produce good-quality virtual native enhancement (VNE) images. The test data set went through initial rejection followed by multiobserver quality control. Rejected test data are available in Figures S1 and S4. *The generative adversarial network (GAN) translating postcontrast cine is specified in Supplemental Material 3. GBCA indicates gadolinium-based contrast agent; and LGE, late gadolinium enhancement.

A total of 912 patients (64±11 years of age; 81% male) with evidence of previous MI on CMR imaging were included in the study. These patients were recruited from the Oxford clinical CMR service (719 patients providing 2585 imaging data set triplets of slice-matched native T1 maps, cine, and LGE short-axis images) and the OxAMI study (193 patients providing 1686 imaging data set triplets; Figure 2). Overall, data from 842 patients (64±11 years of age; 81% male) were included in the training data set and 70 patients (66±11 years of age; 81% male) in the test data set. Patient characteristics, including cardiovascular risk factors, history of revascularization, and age at previous MI, are presented in the Table 1.

**Table 1.**
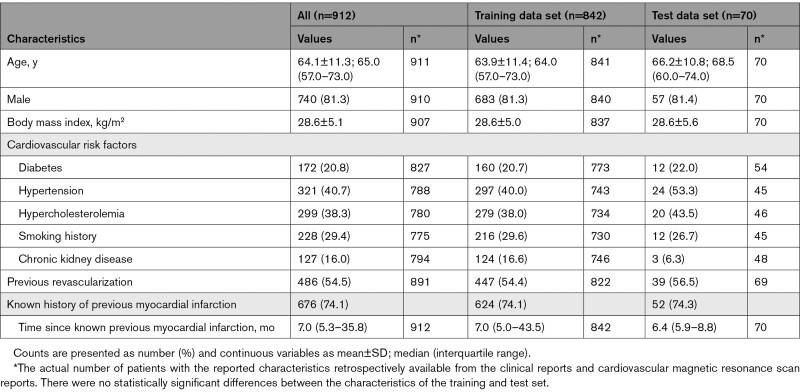
Characteristics of the Patients Whose Cardiovascular Magnetic Resonance Imaging Data Were Used for the Development of Virtual Native Enhancement Deep Learning Models

### CMR Training and Test Data Sets

Of the 912 patients, 842 (3955 image triplets) had postcontrast short-axis cines, which were translated into synthetic precontrast (Supplemental Material 3). These synthetic precontrast cines alongside precontrast T1 maps were used for the training data set. LGE images were also included in the training data set and underwent strict quality control, rejecting any suboptimal quality LGE images, so that the neural networks trained on these materials learned to produce good-quality LGE-like VNE images. In total, 3002 triplets of matching native T1 maps, synthetic precontrast cine, and LGE images (775 patients) were used for training. Seventy separate patients (316 triplets of images) had short-axis cine images acquired before gadolinium contrast injection and were all reserved for the independent test data set to generate VNE images that were completely free of contrast agent. After quality control, 291 triplets (from 68 patients) were output from the independent test data set for multiobserver quality assessment of LGE and VNE before infarct scar quantification (Figure 2).

CMR imaging (with precontrast cines, T1 maps, and LGE) and histopathology data from a porcine model of MI (n=2) were obtained for proof-of-principle demonstration of visuospatial agreement between VNE and LGE against histopathology.

### Image Quality

VNE provided significantly better image quality than LGE, as assessed by all 5 blinded operators. Average quality scores (on the previously described 0- to 100-point scale) were 77.9±5.9 for VNE versus 66.8±4.5 for LGE (n=291; *P*<0.001; Wilcoxon test; Figures S2 and S3). Interobserver variability values were SD=9.82±1.25 and ICC=0.82±0.03. Cases with LGE scored as uninterpretable (11 image slices out of 291) were excluded from further MI assessment, as shown in Figure S4A. An additional patient with previous MI and concomitant substantial valvular heart disease with a severely thinned and dilated left ventricle (3 image slices) was identified during quality control and rejected (Figure S4B). A total of 277 triplets of matching native T1 maps, precontrast cine, and LGE images (66 patients) ultimately were available for the VNE independent testing data set for further scar burden quantification and viability assessment.

### MI Scar Size and Transmurality

**Figure 3.**
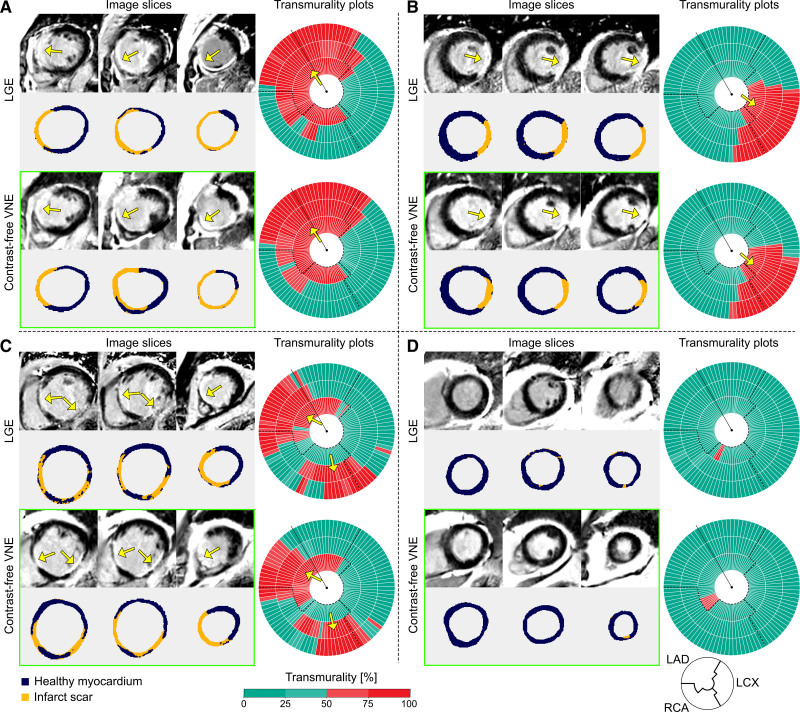
**Examples to illustrate high agreement between LGE and contrast agent–free VNE for the visuospatial distribution and transmurality of myocardial scar. A** through **D**, Three short-axis slices of late gadolinium enhancement (LGE) and virtual native enhancement (VNE) images of the same patient are shown on the left (color masks were used to depict areas of scar as orange and noninfarcted myocardium as dark blue); on the right, scar transmurality measured by LGE and VNE is shown, suggesting the likelihood of myocardial viability (0 to 25%, viable; 26% to 50%, likely viable; 51% to 75%, likely nonviable; 76% to 100%, nonviable). Dashed lines delineate presumed boundaries between myocardial territories. Arrows point to the areas of scar. LAD indicates left anterior descending artery; LCx, left circumflex artery; and RCA, right coronary artery.

Myocardial scar quantification was performed on the independent test data set consisting of 277 short-axis pairs of VNE and conventional LGE images from 66 patients. Four representative patient examples are given in Figure 3A through 3D, showing VNE and LGE images alongside their detected scars (infarcted regions delineated in orange) and transmurality of the scar displayed on bullseye plots for each patient. On bullseye plots, the scar transmurality of each chord was color-encoded into the ranges 0 to 25% (green), 26% to 50% (light green), 51% to 75% (light red), and 76% to 100% (red). These correspond to likelihood of myocardial viability, judged as viable, likely viable, likely nonviable, or nonviable, respectively, per routine clinical practice and landmark studies that showed <10% of segments with >50% infarct transmurality regain function after revascularization.^6^ VNE detected a left anterior descending artery territory myocardial scar in patient A, a left circumflex territory scar in patient B, a multiterritory myocardial scar involving the left anterior descending and right coronary artery territories in patient C, and little scar signal in patient D, all with VNE images closely matching the signals detected by LGE (Figure 3).

**Figure 4.**
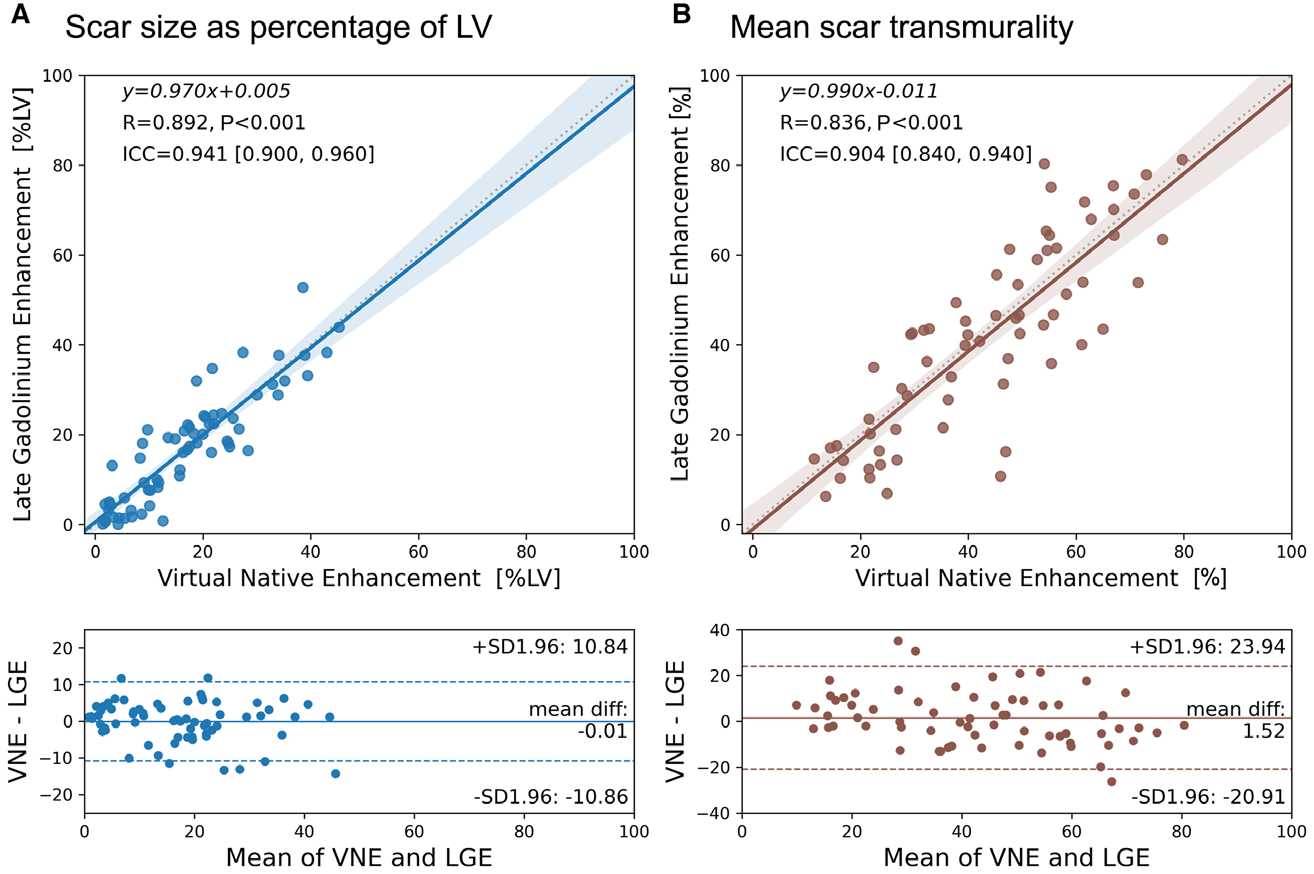
**Assessment of myocardial scar size and transmurality using VNE.** Virtual native enhancement (VNE) correlated strongly with late gadolinium enhancement (LGE) in scar size as a volume fraction of the sampled left ventricular (LV) myocardium (**A**) and in quantifying the mean transmurality of scarred chords per patient (**B**) in 66 test patients. Mean scar transmurality for each patient was calculated by averaging the transmural scar extent across all the chords (using the centerline chord method) that had at least 1% of scar extent on all available slices. Top, Correlation plots with the linear regression equations, Pearson correlation coefficients (R), statistical significance of correlation (*P* value), and intraclass correlation coefficients (ICC; 95% CI shown in brackets) provided. Bottom, Bland-Altman plots to analyze any systematic differences between quantification by VNE and LGE.

In the 66 test patients, VNE showed strong identity correlation with LGE in quantifying myocardial lesion burden for both infarct scar size (Figure 4A; *P*<0.001) and transmurality (Figure 4B; *P*<0.001). The high agreement in both scar size (R=0.89, ICC=0.94) and scar transmurality (R=0.84, ICC=0.90) supports the promise of VNE to replace LGE for noninvasive and contrast-free myocardial scar assessment. Bland-Altman plots (Figure 4) showed excellent accuracy with high precision and no significant bias. The mean differences between VNE and LGE were within ±2% for both scar volume fraction and transmurality, with a 95% confidence interval upper bound of 11% and lower bound of −11% for scar volume fraction and upper bound of 24% and lower bound of −21% for transmurality. The observed differences between VNE and LGE are within the range of reported LGE intramethod variability and interlaboratory inconsistencies.^30^ There was no significant difference in scar assessment between male and female patients using VNE (Figure S5).

### Histopathologic Comparison

**Figure 5.**
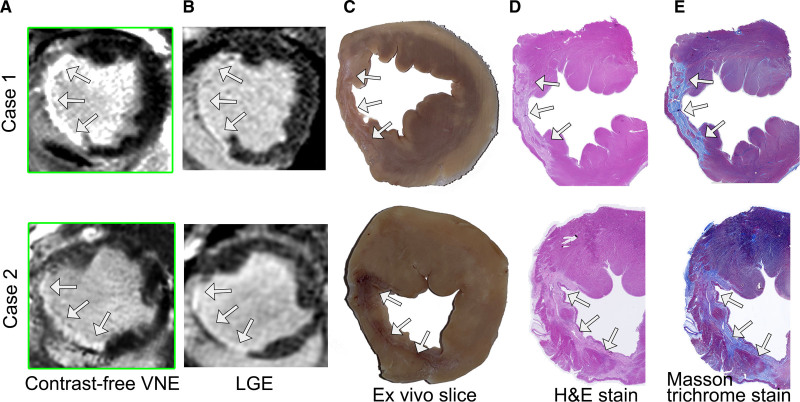
**Histopathologic comparison of VNE on 2 porcine model cases 8 to 9 weeks after myocardial infarction.** Infarction was induced with ligation of the left anterior descending artery for 90 minutes followed by reperfusion. In both cases, virtual native enhancement (VNE) detected chronic myocardial infarction (arrows) in the left anterior descending territory (**A**) and was in high visual agreement with late gadolinium enhancement (LGE; **B**) and the ex vivo pathologic slices. These slices demonstrate macroscopic evidence of infarction (**C**); the infarcted region is pale pink on hematoxylin & eosin (H&E) staining (**D**), with collagen accumulation shown in light blue on Masson trichrome stain (**E**).

Contrast-free VNE images were generated using precontrast cine and native T1 maps from the porcine test data set (n=2) and compared with LGE imaging and slice-matched histopathology. Histopathologic data (macroscopic anatomic specimens, hematoxylin & eosin staining, and collagen accumulation on Masson trichrome stains) were acquired 48 hours after the final CMR scans and matched to the corresponding VNE and LGE images. VNE revealed clear evidence of transmural MI involving the entire left anterior descending artery territory in both cases (Figure 5). The pattern, size, and visual extent of the scars on VNE were in high agreement with those of LGE. Quantitative infarct sizes for the 2 cases were VNE 29.2% versus LGE 30.7% and VNE 25.9% versus LGE 24.8%, respectively. There was excellent visuospatial agreement between the myocardial scar shown on VNE (Figure 5A) and LGE (Figure 5B), macroscopic scarring on anatomic ex vivo slices (Figure 5C), histologic evidence of infarction and fibrosis on hematoxylin & eosin staining (Figure 5D), and collagen accumulation on Masson trichrome staining (Figure 5E).

### Individual Slice Review of the Human Data

Two CMR experts (M.K.B., V.M.F.) independently reviewed the 277 slices of test VNE and LGE images on visuospatial agreement and infarct extent as part of the quality control consensus process. A total of 46 out of 277 (17%) slices had VNE–LGE discrepancies that were attributable to technical factors: position mismatch (29/46 slices), poor native or LGE image quality (11/46 slices), and presence of artefacts (6/46 slices). VNE had excellent agreement on both visuospatial location and scar extent with LGE in the remaining good-quality paired data sets (194/231 slices).

In the remaining 37 out of 231 (16%) slices that were of good quality and well-paired between VNE and LGE, there were true discrepancies in lesion characterization not attributable to the technical factors noted. In 19 of these 37 slices, VNE did not detect small subendocardial LGE-positive areas, potentially because of very thin myocardial walls or limited subendocardial scar extent (ie, very small MIs). In a further 11 slices, VNE–LGE differences were associated with evolving T1 signal abnormalities after recent acute MI: in 3 of these slices, VNE detected more extensive lesions in areas of high myocardial T1 in the absence of LGE (possibly because of residual myocardial edema still present after large acute MIs). In total, there were 4 slices in which VNE lesions had perceivably brighter signal intensity than on the corresponding LGE images. In 8 slices, VNE had missed portions of LGE-positive myocardium in areas of potentially low or pseudonormalized T1 that corresponded to large areas of microvascular obstruction after acute MI. Overall, there were 33 false-negative slices (from 14 individual patients) in which VNE either did not detect small areas of scar or detected them to a lesser extent than shown on LGE. There were no cases where VNE falsely introduced a myocardial scar in LGE-negative slices. Overall, on CMR expert review, VNE demonstrated an accuracy of 84% in detecting a scar when compared with LGE, with a specificity of 100% and a sensitivity of 77%.

## Discussion

This study makes several major contributions. In a large data set from patients with a history of previous MI, assessment of myocardial scars using VNE demonstrated superior image quality compared with LGE CMR, with excellent agreement in visuospatial distribution, scar size, and transmurality (Figures 3 and 4). Proof-of-principle histopathologic comparison showed that VNE had excellent visual agreement with the degree of infarction on macroscopic pathologic specimens and hematoxylin & eosin staining as well as collagen accumulation on Masson trichrome staining (Figure 5). VNE represents a potential paradigm shift in CMR imaging for assessment of myocardial scars, as it can allow significantly faster, lower-cost, and contrast-free CMR scans with improved image quality and similar accuracy to the current gold standard LGE in an easy-to-use format ready for routine clinical use.

The major advantages of VNE are that it obviates the need for gadolinium contrast administration and can substantially shorten scan time. Debates around the safety of gadolinium contrast continue, because studies have shown gadolinium deposition in the brain after repeated administration^31,32^; gadolinium-based contrast agents are also used with caution in certain populations, including those with kidney disease,^33^ which is present in ~20% of patients with MI (Table 1).^34^ Noncontrast imaging shortens scan times and can reduce costs. Others have also recognized the importance of minimizing gadolinium use and applied native T1 mapping to characterize myocardial scars in animal^13^ and human studies of previous MI^12,15–17^; however, this requires complicated quantitative postprocessing image analysis, which is time-consuming and unrealistic for routine clinical practice. Clinical research is increasingly turning to AI-based technologies to address necessary improvements in patient care and clinical throughput, including rethinking contrast enhancement mechanisms in radiology. VNE is a novel solution, given that the technology offers immediate and visually diagnostic representation of T1 maps not previously possible, with excellent agreement with LGE CMR, superior image quality, and good histopathologic correspondence.

Landmark studies using LGE CMR to assess myocardial scars and viability performed histopathologic validation. The spatial extent of late enhancement in a canine model of chronic infarction was shown to match the spatial extent of collagenous scars and replacement fibrosis^35^ and ex vivo LGE CMR accurately quantified and characterized different histologic grades of fibrosis in infarcted swine hearts.^36^ Recent porcine models of MI have incorporated native T1 mapping alongside LGE, providing further validation that these imaging technologies closely correlate with infarction on histopathology, although extent and viability are underestimated if using T1 mapping alone.^14,37^ This may be because T1 values of tissues in vivo are dynamic and can change over time. This is seen in the biology of chronic infarct scars, which can develop lipomatous metaplasia with collagen replacement by interstitial adipocytes and subsequently lower T1 values^38,39^; in acute MI, infarct size and transmurality on LGE may be overestimated because of expansion of the extracellular space from myocardial edema, allowing increased gadolinium contrast uptake.^40–42^ VNE technology provides a solution to this by combining T1 mapping with cine imaging signals containing functional wall motion information, which can improve sensitivity in detecting and quantifying myocardial scars when compared with using T1 mapping on its own. Proof-of-principle demonstration that VNE closely matches the spatial extent of myocardial scars on histopathology in a porcine model of MI, alongside LGE, is also provided.

True discrepancies between VNE and LGE on good-quality and well-paired human data sets provided interesting insights to inform directions for future work. These were primarily attributable to cases in which VNE did not detect small subendocardial scars. This may be caused by very thin myocardium (particularly in the lateral and apical walls), minimal or absent signal on raw T1-weighted images in the presence of normal wall motion, limited subendocardial infarct extent, and better blood pool nulling for LGE acquisitions. VNE detection of small subendocardial scars may be improved by using larger data sets of small, subendocardial scars for machine learning, combined with enhanced blood pool nulling algorithms, and alongside strict quality control procedures and slice selection. VNE–LGE differences may also be explained by evolving T1 signal abnormalities after acute MI, either because of high myocardial T1 (likely reflecting residual edema) or potentially low or pseudonormalized T1 (because of large areas of previous microvascular obstruction, intramyocardial hemorrhage, and development of fatty metaplasia within the scar). Further training on more acute and varied pathologies, as discussed in the following, can overcome these limitations.

There were no cases where VNE falsely introduced a myocardial scar in an LGE-negative patient, but there were 4 examples (from 2 individual patients) where VNE displayed a similar scar location to that on LGE, but with greater extent and higher signal intensity in a blooming-like effect, largely because of enhanced signal abnormalities on T1 maps. Although a scar on LGE is taken as ground truth, in the absence of histopathology in human participants, it is worth considering whether VNE may provide more information than LGE in highlighting the extent of abnormality within the myocardium perhaps not apparent on conventional LGE, given that up to 20% of LGE-negative segments do not show functional recovery despite successful revascularization.^6^ Further work to determine the clinical and functional relevance of such lesions is required.

A robust quality control process is essential for clinical translation of AI technologies to provide confidence that the images generated are reliable to inform clinical decision-making. Here, the incorporation of an automated deep learning quality control algorithm into the data processing pipeline,^27,43^ followed by manual inspection and alongside strict image acquisition standardization processes,^21^ will help reassure the end user of clinical reliability. This may be improved upon using T1 map motion correction strategies.^44^ A further benefit of the VNE technology is that images can be checked in real time on the scanner, allowing the operator to reacquire images if necessary to confirm findings and ensure sufficient image quality without time pressure or concerns about contrast washout. VNE may enable effective screening to determine whether contrast injection is required in a scan before completely replacing LGE, similar to a previous report,^45^ or be combined with gadolinium-free stress CMR for IHD assessment.^46–48^ VNE can thus provide a comprehensive myocardial scar assessment with superior image quality to LGE CMR and quicker throughput (currently ~10 to 15 minutes for a full viability study protocol) than existing rapid IHD protocols.^49^ This may be improved upon with the use of existing accelerated acquisition strategies. A case example of real-world real-time clinical use is given in Supplemental Material 4.

### Limitations

This study has several limitations. VNE technology has been validated in hypertrophic cardiomyopathy^18^ and now for assessment of myocardial scars in patients with a history of previous MI; further work is required to expand VNE to the full spectrum of myocardial pathologies. Patient characteristics associated with the CMR materials for this VNE development were retrospectively collated from historical clinical reports and scan reports, meaning some individual characteristics were not available. Although the close correlation between VNE and LGE may imply similar outcomes on the basis of previous landmark studies using LGE to assess myocardial viability and functional recovery,^6,10^ we have not directly assessed viability and prediction of functional recovery in this study. Patients with acute MI were excluded from this study because of the potential confounding effects of acute myocardial edema, although some patients with MI in this study may still have had residual edema despite being several months out from the acute event. Given that T1 mapping reflects different myocardial tissue properties than LGE and offers additional sensitivity to edema, further development of VNE may allow the differential characterization of acute pathologies beyond LGE, potentially including other edema-sensitive modalities such as T2-based imaging.^50^ Multicenter validation linking VNE to clinical outcomes across a range of pathologies will inform the next steps toward widespread rollout into clinical practice once it is proven to be a reliable clinical tool.

### Conclusions

VNE shows promise as a transformative and paradigm-shifting AI-based technology for the future of CMR imaging. It has superior image quality to conventional LGE, can be applied to clinical practice in a ready and easy-to-use format that is familiar to clinicians, and does not require contrast agents. VNE demonstrates high agreement with LGE CMR and histopathology for the assessment of myocardial scars in cases of previous MI. There is great potential for VNE to reduce scan times and costs, increase clinical throughput, and improve the accessibility of CMR for patients in the near future.

## Article Information

### Sources of Funding

Drs Zhang, Piechnik, Ferreira, and Neubauer acknowledge Oxford BHF Centre of Research Excellence grant RE/18/3/34214. Dr Burrage acknowledges a British Heart Foundation Clinical Research Training Fellowship (FS/19/65/34692). Dr Shanmuganathan acknowledges the Alison Brading Memorial Graduate Scholarship from Lady Margaret Hall, University of Oxford. Dr Gonzales acknowledges support from the Clarendon Fund, University of Oxford. Drs Burrage, Popescu, Myerson, Piechnik, and Ferreira acknowledge National Institute for Health Research Oxford Biomedical Research Centre funding at the Oxford University Hospitals NHS Foundation Trust. Dr Dharmakumar acknowledges funding from the National Institutes of Health/National *Heart, Lung, and Blood Institute* (grants HL133407, HL136578, and HL147133). The OxAMI study is supported by the British Heart Foundation (CH/16/1/32013) and BHF Centre of Research Excellence, Oxford (grant RG/13/1/30181). Drs Ferreira and Channon are supported by the British Heart Foundation Chair award (grant CH/16/1/32013).

### Disclosures

Drs Zhang, Piechnik, Ferreira, and Popescu have authorship rights for patent WO2021/044153 (“Enhancement of Medical Images”; Patent Cooperation Treaty filed in September 2020). Dr Piechnik has patent authorship rights for US patent US20120078084A1 (“Systems and Methods for Shortened Look Locker Inversion Recovery [Sh-MOLLI] Cardiac Gated Mapping of T1”; granted March 15, 2016). Intellectual properties are owned and managed by Oxford University Innovations.

### Supplemental Material

Figures S1–S11

Supplemental Material 2: Extended Deep Learning Method

Supplemental Material 3: Generative Adversarial Network for Synthetic Precontrast Cine

Supplemental Material 4: Case Example of Real-World Clinical Use of the VNE Prototype

## Supplementary Material
