# Supplementary material for: Artificial Intelligence for Contrast-Free MRI: Scar Assessment in Myocardial Infarction Using Deep Learning–Based Virtual Native Enhancement
Source: Circulation. 2022 Sep 20;146(20):1492–503. doi: 10.1161/CIRCULATIONAHA.122.060137 (PMC9662825; doi:10.1161/CIRCULATIONAHA.122.060137)
Supplement: Supplementary file 1 [file cir-146-1492-s001.pdf]

# SUPPLEMENTAL MATERIAL

## Artificial Intelligence for Contrast-free MRI: Scar Assessment in Myocardial Infarction Using Deep Learning-based Virtual Native Enhancement (VNE)

Qiang Zhang[†], Matthew K Burrage[†], Mayooran Shanmuganathan, Ricardo A Gonzales, Elena Lukaschuk, Katharine Thomas, Rebecca Mills, Joana Leal Pelado, Chrysovalantou Nikolaidou, Iulia A Popescu, Yung P Lee, Xinheng Zhang, Rohan Dharmakumar, Saul G Myerson, Oliver Rider, Oxford Acute Myocardial Infarction (OxAMI) Study, Keith M Channon, Stefan Neubauer, Stefan K Piechnik*, Vanessa M Ferreira*

[†] Qiang Zhang and Matthew K Burrage contributed equally as joint first authors

* Vanessa M Ferreira and Stefan K Piechnik contributed equally as joint senior authors

Correspondence: Qiang Zhang, Oxford Centre for Clinical Magnetic Resonance Research (OCMR), Level 0, John Radcliffe Hospital, Headington, Oxford, United Kingdom, OX3 9DU. Email: qiang.zhang@cardiov.ox.ac.uk, Tel: +44 (0) 7442336388

# Supplemental Material I:

# Supplemental Figures

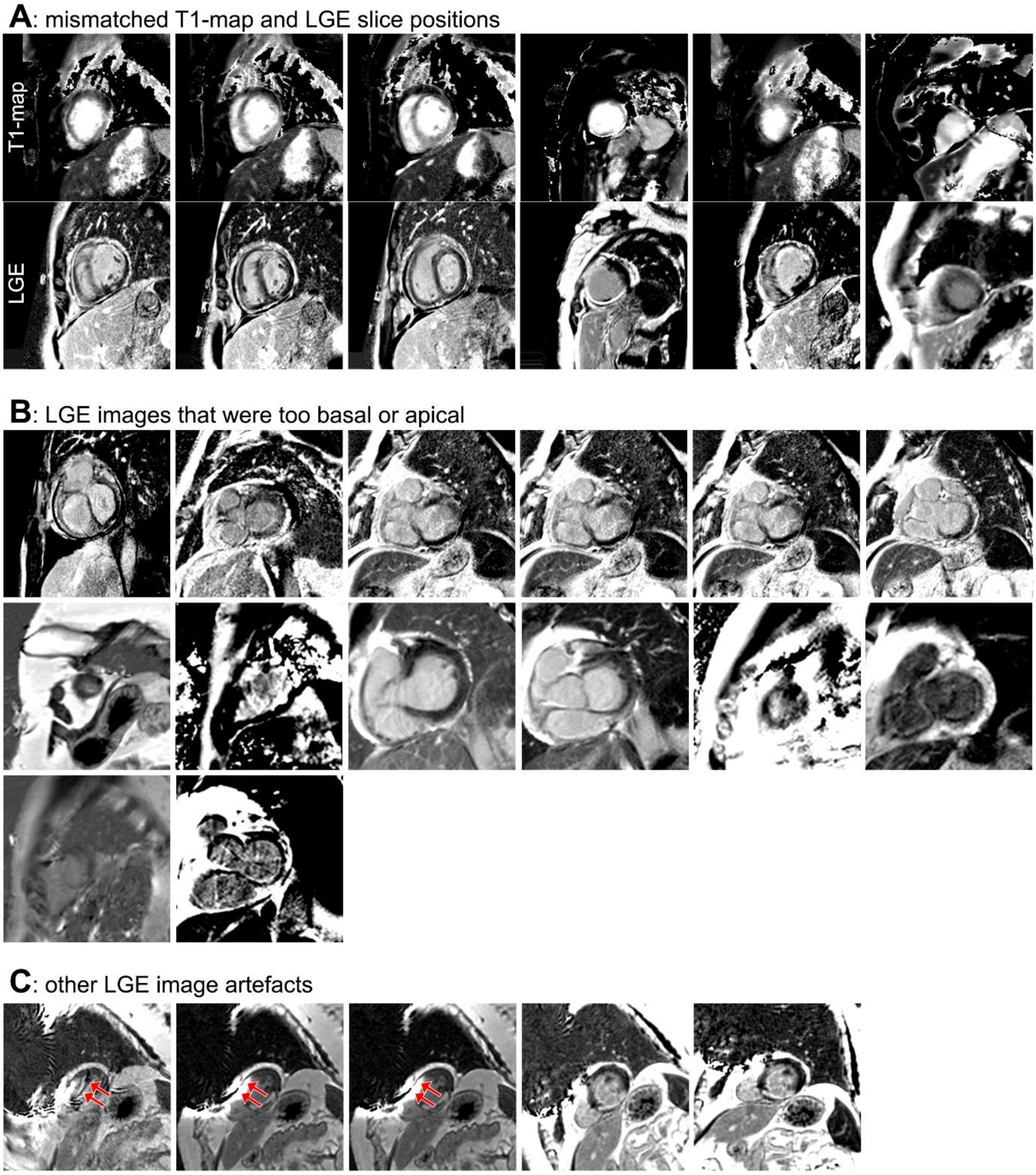


**Figure S1. Test materials that were rejected. A**: 6 image slices were excluded due to mismatch between T1-maps (TOP, shown in grey scale) and LGE (BOTTOM). The mismatched slice positions were evident by different right ventricle shapes and positions. **B**: Fourteen image slices were excluded for slice position being too basal or apical. **C**: One patient (5 image slices) was rejected for other LGE image artefacts (red arrows).

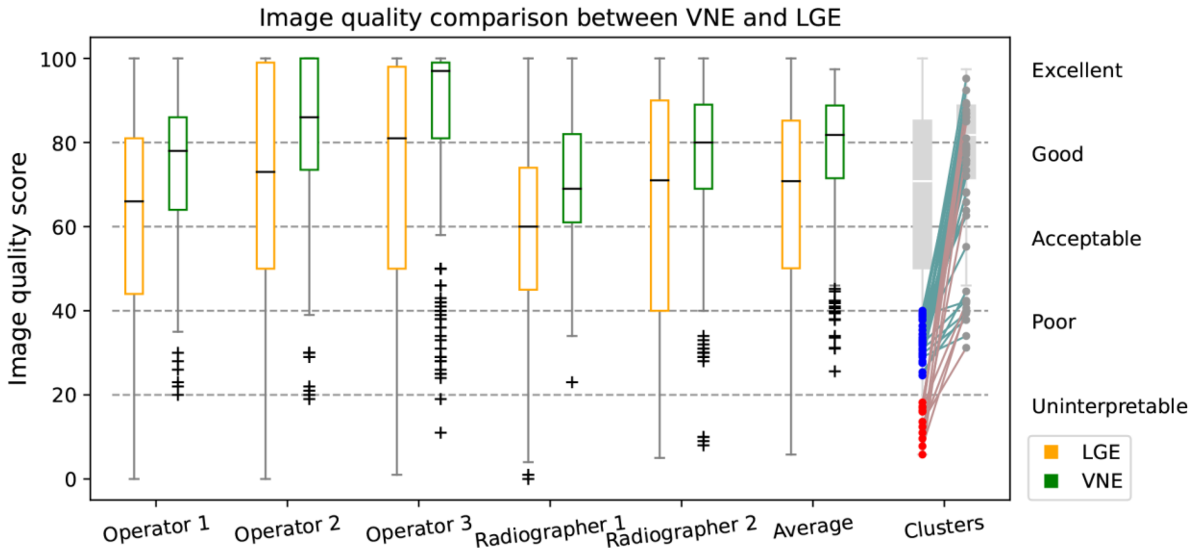


**Figure S2. Image quality assessment on 291 pairs of VNE and LGE images test materials (68 patients).** VNE provided significantly better image quality, assessed independently by five blinded operators and their average scores (all p<0.001). VNE provided superior imaging quality in all cases with “uninterpretable” (red clusters) or “poor” (blue) LGE images.

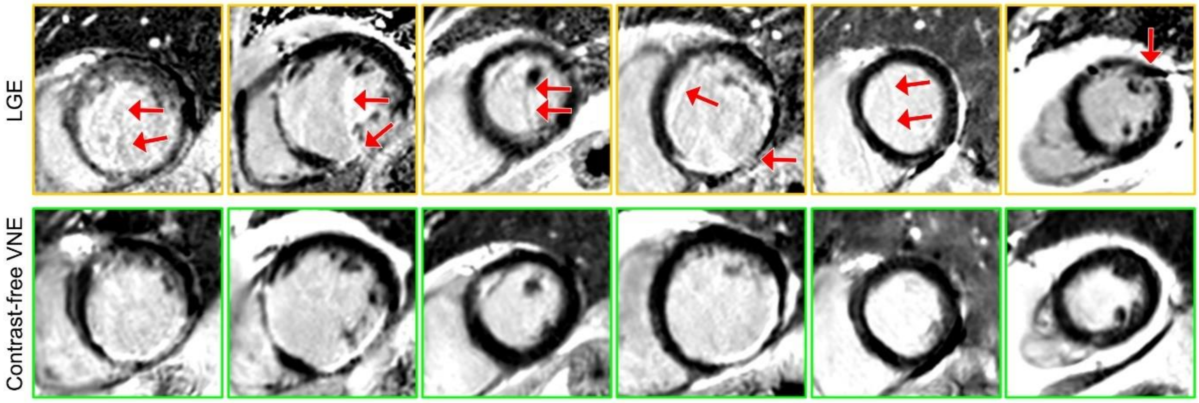


**Figure S3. Examples to demonstrate better image quality of VNE than LGE.** Conventional LGE can be affected by inaccurate inversion time (TI) selection and breathing artefacts attributable to patient fatigue at the last stage of long scanning sessions. Arrows point to the LGE artefacts. VNE provided better and more consistent image contrast and quality overall.

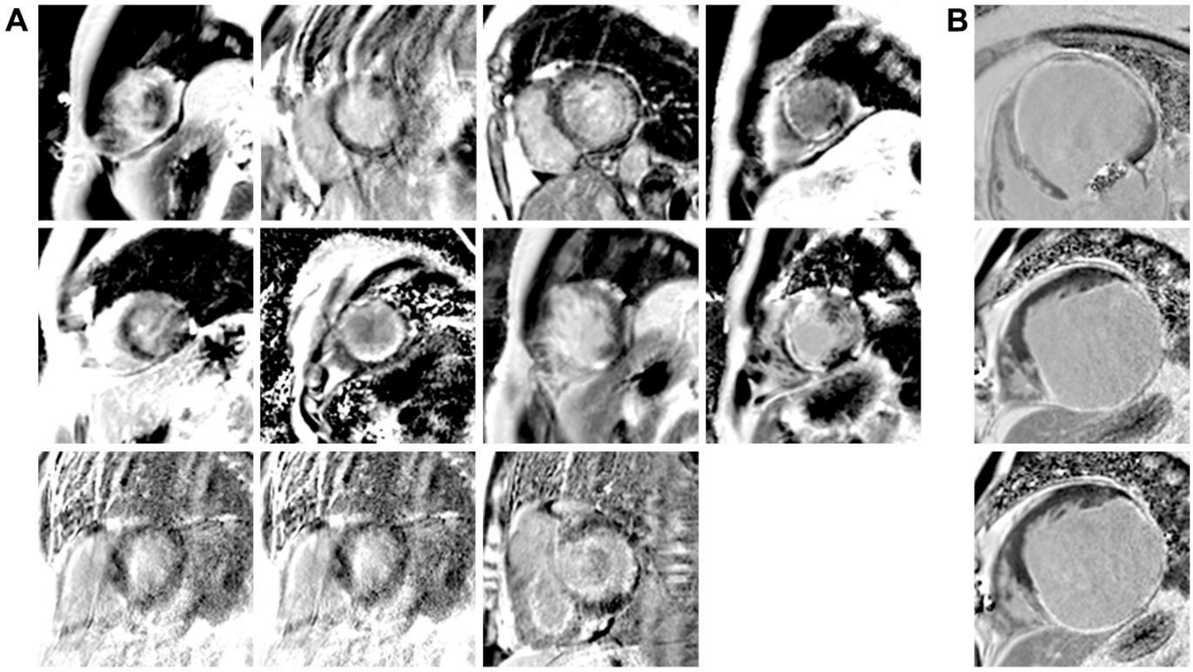


**Figure S4. Test materials that were excluded after multi-observer quality control. A:** 11 slices of LGE with uninterpretable image quality. **B:** 3 slices of LGE from a patient with MI and concomitant significant valvular disease and a severely thinned and dilated left ventricle.

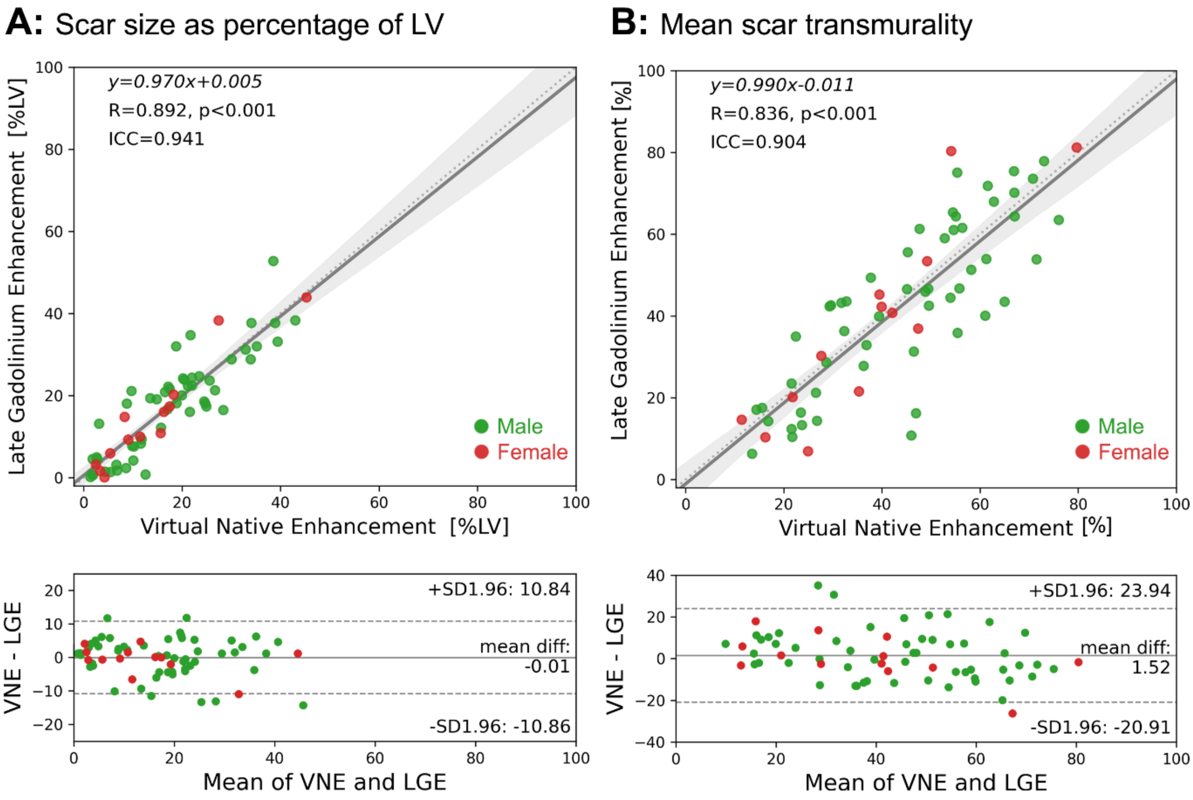


**Figure S5.** Myocardial scar assessment on males (green) and females (red). There was no significant difference between male and female patients in the assessment of myocardial scar size (A) and transmurality (B) using VNE.

## Supplemental Material II:

# Extended Deep Learning Method

This supplemental material provides the deep learning method details for reproducibility of the virtual native enhancement (VNE) technology for myocardial viability and infarction assessment in cardiovascular magnetic resonance (CMR). The VNE image generator was constructed based on multiple streams of U-nets, using short-axis cines and native T1-mapping data, and trained using the conditional generative adversarial network (cGAN) strategy.

## VNE image generator

U-Net is a popular generative convolutional neural network (CNN) architecture that translates an input image into corresponding output masks or images. The VNE generator in this development consists of two streams of 14-layer adapted U-Net blocks (**Figure S6**). One stream utilizes magnitude cine images with 25 channels representing time frames. The other stream processes T1-mapping data with 8 channels representing 7 inversion recovery-weighted magnitude images and 1 reconstructed T1-map, stacked together for computing efficiency. The final convolutional layer of these U-Nets was removed, such that the second last layer of each stream outputs feature maps of 64 channels. All feature maps are concatenated and passed onto a further 6-layer U-Net block (**Figure S7**) to fuse the information and produce a single-channel VNE image.

Inputs (H×W)

| Name | Kernel size | Filters | Stride | Input size | Output size | BN | Dropout | Activation |
|---|---|---|---|---|---|---|---|---|
| Conv2D_1 | 4x4 | 32 | 1 | - | H×W×32 | No | No | LeakyReLU |
| Conv2D_2 | 4x4 | 64 | 2 | H×W×32 | H/2×W/2×64 | Yes | No | LeakyReLU |
| Conv2D_3 | 4x4 | 128 | 2 | H/2×W/2×64 | H/4×W/4×128 | Yes | No | LeakyReLU |
| Conv2D_4 | 4x4 | 256 | 2 | H/4×W/4×128 | H/8×W/8×256 | Yes | No | LeakyReLU |
| Conv2D_5 | 4x4 | 512 | 2 | H/8×W/8×256 | H/16×W/16×512 | Yes | No | LeakyReLU |
| Conv2D_6 | 4x4 | 512 | 2 | H/16×W/16×512 | H/32×W/32×512 | Yes | No | LeakyReLU |
| Conv2D_7 | 4x4 | 512 | 2 | H/32×W/32×512 | H/64×W/64×512 | Yes | No | LeakyReLU |
| | | | | | | | | |
| Upsample | - | - | - | H/64×W/64×512 | H/32×W/32×512 | - | - | |
| Conv2D_8 | 4x4 | 512 | 1 | H/32×W/32×512 | H/32×W/32×512 | Yes | Yes | LeakyReLU |
| Upsample | - | - | - | [H/32×W/32×512]×2 | H/16×W/16×1024 | - | - | |
| Conv2D_9 | 4x4 | 512 | 1 | H/16×W/16×1024 | H/16×W/16×512 | Yes | Yes | LeakyReLU |
| Upsample | - | - | - | [H/16×W/16×512]×2 | H/8×W/8×1024 | - | - | |
| Conv2D_10 | 4x4 | 256 | 1 | H/8×W/8×1024 | H/8×W/8×256 | Yes | No | LeakyReLU |
| Upsample | - | - | - | [H/8×W/8×256]×2 | H/4×W/4×512 | - | - | |
| Conv2D_11 | 4x4 | 128 | 1 | H/4×W/4×512 | H/4×W/4×128 | Yes | No | LeakyReLU |
| Upsample | - | - | - | [H/4×W/4×128]×2 | H/2×W/2×256 | - | - | |
| Conv2D_12 | 4x4 | 64 | 1 | H/2×W/2×256 | H/2×W/2×64 | Yes | No | LeakyReLU |
| Upsample | - | - | - | [H/2×W/2×64]×2 | H×W×128 | - | - | |
| Conv2D_13 | 4x4 | 32 | 1 | H×W×128 | H×W×32 | Yes | No | LeakyReLU |

Concatenate → Feature maps (H×W×64)

**Figure S6. Configuration of the convolutional stream.** The convolutional stream is used to extract feature maps from a native modality such as cine or T1-mapping. BN = batch normalization. Dropout rate was set as 0.2.

Feature maps ([H×W×64]×2)

| Name | Kernel size | Filters | Stride | Input size | Output size | BN | Dropout | Activation |
|---|---|---|---|---|---|---|---|---|
| Conv2D_1 | 4x4 | 64 | 1 | [H×W×64]×2 | H×W×64 | Yes | No | LeakyReLU |
| Conv2D_2 | 4x4 | 128 | 2 | H×W×64 | H/2×W/2×128 | Yes | No | LeakyReLU |
| Conv2D_3 | 4x4 | 256 | 2 | H/2×W/2×128 | H/4×W/4×256 | Yes | No | LeakyReLU |
| | | | | | | | | |
| Upsample | - | - | - | H/4×W/4×256 | H/2×W/2×256 | - | - | |
| Conv2D_4 | 4x4 | 128 | 1 | H/2×W/2×256 | H/2×W/2×128 | Yes | No | LeakyReLU |
| Upsample | - | - | - | [H/2×W/2×128]×2 | H×W×256 | - | - | |
| Conv2D_5 | 4x4 | 64 | 1 | H×W×256 | H×W×64 | Yes | No | LeakyReLU |
| Conv2D_6 | 4x4 | 1 | 1 | [H×W×64]×2 | H×W×1 | No | No | Tanh |

VNE

**Figure S7. Configuration of the CNN fusion block.** The fusion block combines feature maps of multiple native modalities and derive a VNE image.

# VNE generator training using cGAN

cGAN consists of two models trained together in an adversarial manner: a generative model *G* and a discriminative model *D*. *G* is the VNE generator that produces the VNE images which resembles LGE. In this application, we use two discriminators *D1*, *D2* in the discriminator models, where *D1* is a classification neural network (**Figure S8 A**) that encourages VNE to look like LGE images, and *D2* (**Figure S8 B**) encourages the low-level image clarity of VNE to match good quality cine images, therefore achieving higher signal to noise ratio of VNE. *G* and *Ds* are trained simultaneously.

## Objective

*G* and *Ds* are trained by optimizing the value of an objective function. Suppose there is a native CMR input $x$ which is processed by *G* to produce the VNE image $G(x)$ that resembles the LGE image $y$ and matches good quality cine $z$ in image clarity. In this application, the objective for cGAN optimization can be expressed as an adversarial minimax game:

$$\min_{G} \max_{D1,D2} \Big(\lambda_1 \|y - G(x)\|_1 + \lambda_2 \|VGG(y) - VGG\big(G(x)\big)\|_1 + \log\Big(1 - D1\big(G(x)\big)\Big) + \log D1(y) + \log\Big(1 - D2\big(G(x)\big)\Big) + \log D2(z)\Big),$$

where *G* is optimized to minimize the objective function, while *D1* and *D2* are optimized to maximize the objective function. The first term is an *L1* loss that encourages the generator *G* to produce $G(x)$ (optionally including intermediate VNE signals produced at the end of cine and T1-map streams) that matches $y$ pixel by pixel. Rather than exact replication of real LGE signal intensities, this VNE application focuses on enhancing the native CMR signals and translating the native images into the presentation of LGE. To account for this, the second term is a perceptual loss which calculates differences between high-level image feature representations of $G(x)$ and $y$. The features, denoted by $VGG\big(G(x)\big)$ and $VGG(y)$, are generated from the last convolutional layer of a 16-layer VGG network pre-trained on ImageNet. In the third and fourth terms, $G(x)$ and $y$ are input to the discriminator *D1* which produces the "realness" labels $D1(y)$ and $D1\big(G(x)\big)$ as 1: "real" LGE or 0: "virtual" LGE. The objective of training *D1* is to distinguish between real and virtual LGE images, i.e., to maximize the two terms. In the last two terms, image patches were randomly sampled from images $G(x)$ and $z$ and input to the discriminator *D2*. The objective of training *D2* is to distinguish between VNE and cine patches based on the low-level feature clarity. Simultaneously, *G* is encouraged to produce VNE that cannot be distinguished from real

LGE appearance by the discriminator *D1*, and from cine image clarity by *D2*, i.e., to minimize the third and fifth terms. The weighting parameters $\lambda_1$ and $\lambda_2$ are used to balance the magnitude of terms. In this application, a much lower $\lambda_1 = 20$ and higher $\lambda_2 = 200$ were set in order to enforce matching in perceptual features rather than pixel values. The strategy results in a trained generator that translates the existing native CMR signals into LGE image appearance with improved image clarity.

To account for inevitable position differences between the native modalities and LGE used in training, an additional modification was added to the first L1 loss term, to shift the LGE image locally and search for the best match:

$$\min_{i,j} \left\| y_{i,j} - G(x) \right\|_1,$$

where $i, j \in \{-10, -9 \ldots, 10\}$ denote the shift in pixels horizontally and vertically.

## Training and optimization specifications

To improve the robustness of the model, on-the-fly augmentation was employed on the training dataset, introducing uniformly distributed random rotation in 360 degrees, zoom in range 0.8 to 1.0, shear within the range of 0.2, and translation within 4 pixels around the manually annotated center of the LV cavity, using Python Keras packages. The specifications of training CNN were: input size=192x192, batch size=16, learning rate=2e-4; Adam was used as the optimizer. The networks were implemented in TensorFlow and trained using an NVIDIA GeForce RTX 3090 GPU, for 400 epochs, taking approximately 24 hours.

**A**

VNE or LGE (H×W)

| Name | Kernel size | Filters | Stride | Input size | Output size | BN | Activation |
|---|---|---|---|---|---|---|---|
| Conv2D_1 | 3x3 | 64 | 1 | H×W×1 | H×W×64 | No | LeakyReLU |
| Conv2D_2 | 3x3 | 128 | 2 | H×W×64 | H/2×W/2×128 | No | LeakyReLU |
| Conv2D_3 | 3x3 | 128 | 1 | H/2×W/2×128 | H/2×W/2×128 | No | LeakyReLU |
| Conv2D_4 | 3x3 | 256 | 2 | H/2×W/2×128 | H/4×W/4×256 | No | LeakyReLU |
| Conv2D_5 | 3x3 | 256 | 1 | H/4×W/4×256 | H/4×W/4×256 | No | LeakyReLU |
| Conv2D_6 | 3x3 | 512 | 2 | H/4×W/4×256 | H/8×W/8×512 | No | LeakyReLU |
| Dense | - | - | - | H/8×W/8×512 | 1024 | No | LeakyReLU |
| Dense | - | - | - | 1024 | 1 | No | Linear |

"Realness" score

**B**

VNE or LGE (H×W)

| Name | Kernel size | Filters | Stride | Input size | Output size | BN | Activation |
|---|---|---|---|---|---|---|---|
| Conv2D_1 | 3×3 | 32 | 1 | 32×32×1 | 32×32×32 | No | LeakyReLU |
| Conv2D_2 | 3×3 | 64 | 2 | 32×32×32 | 16×16×64 | No | LeakyReLU |
| Conv2D_3 | 3×3 | 64 | 1 | 16×16×64 | 16×16×64 | No | LeakyReLU |
| Conv2D_4 | 3×3 | 128 | 2 | 16x16×64 | 8×8×128 | No | LeakyReLU |
| Conv2D_5 | 3×3 | 128 | 1 | 8×8×128 | 8×8×128 | No | LeakyReLU |
| Conv2D_6 | 3×3 | 256 | 2 | 8×8×128 | 4×4×256 | No | LeakyReLU |
| Dense | - | - | - | 4×4×256 | 512 | No | LeakyReLU |
| Dense | - | - | - | 512 | 1 | No | Linear |

Texture score

**Figure S8. Configuration of the neural network discriminators used in the conditional Generative Adversarial Network training approach. A:** Discriminator *D1* to encourage VNE to look like LGE; **B:** *D2* to encourage VNE to resample cine clarity, therefore achieve higher signal to noise ratio.

## Supplemental Material III:

# Generative Adversarial Network for Synthetic Pre-contrast Cine

For CMR scans in clinical practice, short-axis cines are typically acquired post-contrast as per routine imaging protocols. We translated post-contrast cines into synthetic pre-contrast images for training the contrast-free VNE technology, using a dedicated conditional generative adversarial network (cGAN).

To train the cGAN model, 328 CMR scans that had both pre-contrast cines (usually acquired during the pilots and planning, **Figure S9 A**) and post-contrast cines were identified from the clinical dataset from Oxford. The scans were randomly split into a training dataset of 295 scans (315 pairs of pre- and post-contrast cines with matched slice positions), and a validation dataset of 33 scans (37 pairs of cines). Each cine has 25 temporal frames, which gives a total of 7875 images for training, and 925 images for validation.

The Generator uses the same architecture as in **Figure S6**, with a convolutional layer added at the end (4x4 kernel size, 1 filter, Tanh activation) to produce the cine images. The Discriminator has the same architecture as in **Figure S8 A**. The training procedure is illustrated in **Figure S9 B**, where the Generator learned to produce synthetic pre-contrast cine images that have the same pixel values (trained with L1 loss) and similar image appearance (trained with the Discriminator). The Discriminator was trained simultaneously to distinguish between true and synthetic pre-contrast cine images in an adversarial manner. The dedicated cGAN for generating synthetic pre-contrast cine was trained and optimized in the same manner as the cGAN for VNE (Supplemental Material II). Illustrative examples of synthetic pre-contrast cine frames produced by the trained Generator are given in **Figure S10**.

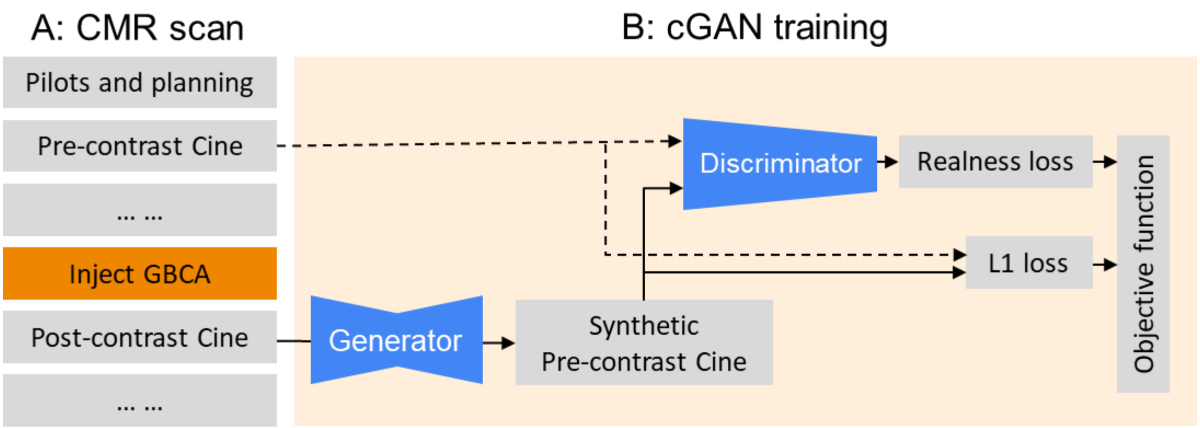


**Figure S9. Training procedure of a cGAN model to produce synthetic pre-contrast cine images. A:** a CMR scan with paired pre- and post-contrast cines. **B:** cGAN training which encourage the Generator to produce synthetic pre-contrast cines that have similar appearance and pixel values with the real pre-contrast cines.

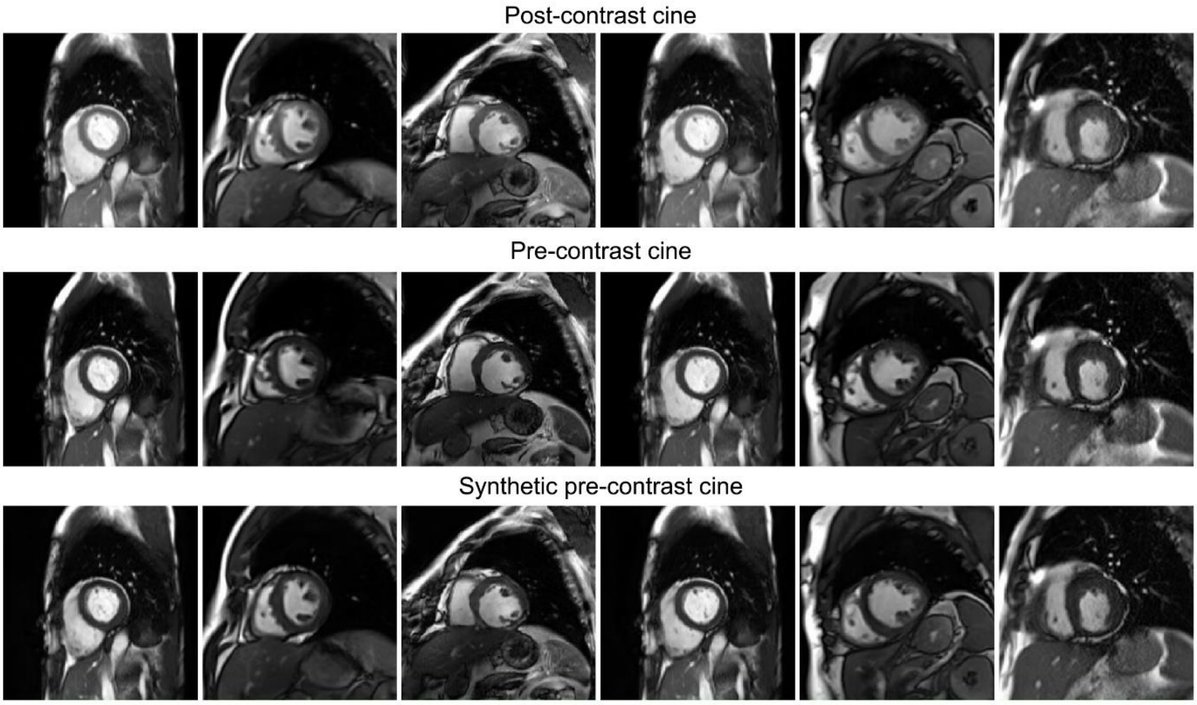


**Figure S10: Illustrative examples of synthetic pre-contrast cine frames from the test set presented together with the matched post-contrast and real pre-contrast cines.**

## Supplemental Material IV:

# A case example of real-world clinical use of the VNE prototype

A 58-year-old man, with a previous ST elevation myocardial infarction (STEMI) treated with primary percutaneous coronary angiography (PCI) to the left anterior descending (LAD) artery, was referred for CMR to investigate recent episodes of chest pain. CMR was performed on a 1.5T MR system (Siemens Avanto FIT) (**Figure S11 A**). Cine imaging and native T1-mapping (ShMOLLI method) were performed before GBCA administration, with immediate generation of VNE images before post-contrast LGE acquisition (**Figure S11 B**). VNE images compared closely to the LGE images, showing a near-transmural myocardial infarction in the mid-distal anterior wall, anterior septum and apex, with little viability (**Figure S11 C**). This real-world, real-time case example displays high agreement between VNE and LGE, with superior image quality of VNE when compared to LGE (breathing motion artefact at the end of the scan session).

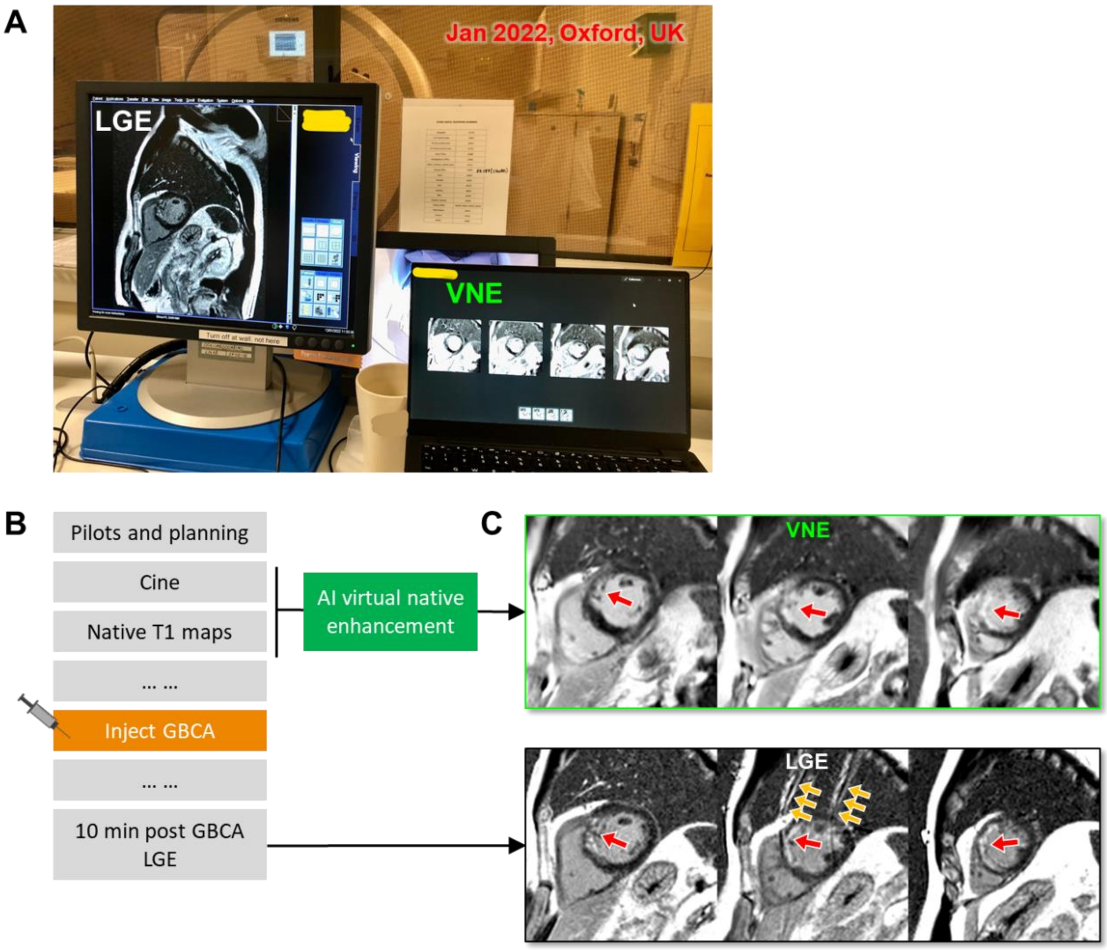


**Figure S11: A case example of real-world clinical use of the VNE prototype. A:** A photo taken on the site. **B:** CMR protocol. **C:** The produced VNE images in comparison with LGE.
